# Proximate determinants of fertility in Ethiopia; an application of revised Bongaarts model

**DOI:** 10.1186/s12978-019-0677-x

**Published:** 2019-02-04

**Authors:** Tariku Laelago, Yitagesu Habtu, Samuel Yohannes

**Affiliations:** 1 School of Public Health, Wolaita Sodo University, Wolaita Sodo, Ethiopia; 2 Department of health information technology, Hossana College of Health Science, Hossana, Ethiopia; 3 Department of midwifery, Hossana College of health sciences, Po. Box 159, Hossana, Ethiopia

**Keywords:** Bongaarts, Fertility, Proximate, region, Education, Wealth index, Residence

## Abstract

**Background:**

World population is growing at about 80 million people each year. Ethiopia is the 12th most populous country in the world. Existing literatures showed that the role of proximate determinants in inhibiting the total fertility has not yet been determined from the DHS data in the country. This study may provide evidence based information regarding the observed changes in total fertility. The objective of this study was assessing proximate determinants of fertility and the role of selected socio-economic variables in influencing fertility in Ethiopia.

**Methods:**

The EDHS data of 2011 and 2016 were used in our study. A total of 16,515 eligible women included in 2011 and 15,683 in 2016 surveys made up the sample for the study. The roles of each of the four proximate determinants in declining fertility have been determined. The background variables selected for the analysis include: region of residence, educational status, wealth index and place of residence. The Bongaart model is used to explain the observed socio-economic differentials in fertility during the two survey years.

**Results:**

In 2011, index of marriage inhibited fertility by 37.8%, however in 2016 it inhibited fertility by 34.4%. In 2011, contraceptive use reduced fertility by 28.5% while in 2016 it reduced fertility by 30.7%. The index of postpartum infecundity decreased fertility by 34.7% in 2011 and by 34.5% in 2016. Foetal wastage inhibited fertility by 9.2% in both survey years. The total fertility rate in 2016 was 4.14 whereas the projected total fertility in 2020 will be 3.2 children per woman.

**Conclusion:**

Among the four proximate determinants of fertility, the contribution of index of marriage was the highest in inhibiting fertility in 2011. On the other hand, the contribution of postpartum infecundability was the highest in inhibiting fertility in 2016. The contribution of the index of contraceptive in inhibiting fertility increased from 28.5% in 2011 to 30.7% in 2016. The index of foetal wastage contributed the least in both 2011 and 2016 survey years. Therefore, strategies have to be designed to promote the contraceptive use and breast feeding practices among the reproductive women.

## Plain English summary

World population is growing. The Ethiopia population is also increasing. Fertility rate is an important determinant of population size. In this study the contribution of the proximate determinants of fertility has been determined.

A total of 16,515 (2011EDHS) and 15,683 women (2016 EDHS) were the study participants. The extent to which each of the four proximate determinants contributed in declining the total fertility has been determined. The Bongart revised model was used to explain the observed socio-economic differentials in fertility.

In 2011, 37.8% of births were inhibited due to the effect of delayed marriage & non marriage, whereas its contribution in declining fertility was 34.4% in 2016. The influence of contraception in fertility inhibition was 28.5 and 30.7% below its biological maximum in 2011 and 2016 respectively. Postpartum infecundability decreased the total fertility by 34.7% in 2011 and 34.5% in 2016. The effects of index of foetal wastage were the same i.e. it inhibited only 9.2% of the total births in both survey years. Based on our projection the future total fertility of Ethiopia in 2020 will be 3.2.

In summary, index of marriage and postpartum infecundability were the leading fertility inhibiting factors in 2011 and 2016 respectively. The contribution of the index of contraceptive in inhibiting fertility increased from 28.5% in 2011 to 30.7% in 2016 whereas foetal wastage contributed the least in both survey years. Therefore, contraceptive use and breast feeding practices have to be promoted among the study subjects.

## Background

In demography, fertility indicates the product or output of reproduction, rather than the ability to have children. The physiological ability to have children that is manifested roughly in the period between menarche and menopause in women is termed fecundity. Demographers define a third, further aspect of reproduction, fecundability, which is the probability of becoming pregnant, or the likelihood of exposure to the possibility, that depends on the pattern of sexual and pregnancy preventive behaviours [1].

Demographically observed fertility or infertility is the result of a well-defined number of both biological and behavioural factors, which serve to mediate the influence of culture, society, economic conditions, living standards, and other similar background determinants on individual reproductive behaviour. Together, these biological and behavioural factors are called the proximate determinants of fertility and they are the factors through which, and only through which, the social and economic environment can influence individual procreation. Together, these factors constrain fertility, and explain why women do not have the maximum possible number of children, which would be about 35 if they reproduced continuously from, say, the age of 18, to about the age of 45, having a birth every 9 months [1].

World population is growing at about 80 million people each year. If current trends keep on, there will be about 9.2 billion people on this planet by the mid of 21st century. Virtually all of the major problems that threaten the world today are connected with rapid population growth. It fuels health risk, hunger or poverty, water scarcity, environmental depletion and political instability [2]. Ethiopia is the 12th most populous country in the world [3] with a recently estimated total fertility rate [TFR] of 4.6 children per woman [4].Though, Ethiopia has achieved remarkable declines in fertility rate, the changes are not shared equally among the different administrative regions of the country [5]. Some regions have shown a fall in fertility, whereas some are moving to the opposite direction and the other regions still have shown no change in total fertility rate [6].

The TFR of Ethiopia declined from 5.5 children per woman in 2000 to 4.6 children per woman in 2016 [4, 7]. This puts the country at the rank of 14th highest total fertility rate in the world [4]. The TFR among women declined from 6.0 children in 2000 to 5.2 children in 2016 and 3.0 children in 2000 to 2.3 children in 2016 in rural and urban areas respectively [4, 7].

Studies done in different countries identified various diffentials of fertility. Study conducted in Bangladesh indicated contraceptive practice as a key factor in fertility change [8]. In Zambia, marriage and postpartum infecundity accounted for the largest inhibiting effect on natural fertility from its biological maximum of 19.10 [9]. In Philippines**,** fertility is differentiated by ethnic group, place of residence, educational attainment, women’s work and age at marriage. Contraceptive use and wealth index also played an important role in explaining the fertility differentials [10]. The findings from Ghana indicated a woman’s contraceptive behavior; marriage status and postpartum infecundability as important predictors of fertility outcomes. Higher education levels and urban residence were consistently associated with lower fertility rates [11]. Most of the decline in fertility in Sub-Saharan Africa is attributable to increases in the proportions of women unmarried and to a lesser extent increases in contraceptive use [12]. Study done in Sub-Saharan Africa, identified proximate determinants of fertility such as non-marriage, post-partum amenorrhea and contraceptive use as an important factors in inhibiting fertility. The Study also identified the background variables such as desired number of children, national family planning effort, under-five mortality, level of education, female participation in the workforce, and place of residence as influencing factors of fertility [13]. In Asian countries, marriage pattern and induced abortion played an important role in reducing fertility among women [14].

John Bongaarts early framed a model explaining fertility as a function of proximate determinants of fertility through which the background variables affect fertility [15]. Revised version of John Bongaarts’ model proposed by John Stover [16] is currently in use to indicate the roles of the proximate determinants in predicting the TFR [17]. Our research used a revised Bongaarts model to explain the contribution of each proximate determinant in the total change of fertility. These proximate determinants include exposure to childbearing (marriage, consensual union and sex), contraceptive use, total foetal wastage and postpartum infecundity [16]. Getting abortion data is difficult in Ethiopia. Therefore, we used the revised Bongaarts model to include foetal wastage instead of abortion. The total fertility as a key demographic indicator has been declining. But the contribution of various reproductive behaviours and background variables to this change need to be timely assessed. This helps to determine how much they have inhibited fertility rate over time. The role of background factors in influencing the proximate determinants of fertility and the extent to which proximate determinants influence the fertility decline has to be investigated from the most recently available database. The four background variables: region of residence, educational status, wealth index and place of residence were preferred to use based on their effect on influencing fertility [8–15]. Existing literatures showed that the role of proximate determinants in inhibiting the total fertility has not yet been determined from the DHS data in the country. This study may provide evidence based information regarding the observed changes in total fertility between the two recent DHS years, EDHS 2011 and 2016, and the independent contribution of the proximate determinants to these changes. It also furnishes useful baseline information for further studies which will prospectively make use of the EDHS database. Therefore, the objective of this study was assessing proximate determinants of fertility and the role of selected socio-economic variables in influencing fertility in Ethiopia.

## Methods

### Data sources

The source of data for our study was the EDHS conducted in 2011 and 2016. A total of 16,515 eligible women enrolled in 2011 and 15,683 in 2016 surveys made up the sample for this study. The roles of each of the four proximate determinants in fertility decline was determined. One important fertility determinant variable was not captured by these datasets namely abortion due to lack of appropriate data. Instead of abortion data, foetal wastage data was included in the analysis. The selected background variables include: region of residence, educational status, wealth index and place of residence.

### Data analysis methods

John Bongaarts framework for analysing proximate determinants of fertility model was used to analyse the data. The four proximate determinants of fertility, total fertility and background variables were calculated by using Microsoft excel software. In the following section we presented detailed analysis of fertility differential model that quantifies the negative and positive effects of each of the socio-economic and cultural factors on fertility through various intermediate fertility variables. For illustration, the model was used to explain the observed socio-economic differentials in fertility during the 2011 and 2016 EDHS.

### Bongaarts fertility differential model

Bongaarts model of estimating the effect of four proximate determinants assumes that the natural reproductive capacity that is total fecundity rates (TF) is nearly the same for all women, but their actual reproductive performance is modified by four major proximate determinants. In his model [7], Bongaarts expressed TFR as the product of four indices measuring the fertility inhibiting effect of these four indices and is the average number of live births expected among women who during their entire reproductive period, remain married, do not use contraception, do not have any induced abortion and do not breastfeed their children [17]. According to Bongaarts model, the TFR can be written as

1 $$ \mathrm{TFR}=\mathrm{CmxCcxCaxCiXTF} $$

Where, C**m** is the index of proportion married, C**c** is the index of non-contraception, C**a** is the index of induced abortion, C**i** is the index of lactational infecundability and TF is the total fecundity.

### Revised Bongaarts model

This revised model replaced only the index of all foetal wastage (sum of miscarriage, abortion and still birth) instead of the index of abortion. Other three indices have remained the same as proposed by Bongaarts original model [18].

The revised Bongaarts model [8] is represented as

2 $$ \mathrm{TF}\mathrm{R}=\mathrm{Cm}\times \mathrm{Cc}\times \mathrm{Cfw}\times \mathrm{Ci}\times \mathrm{TF} $$

TFR is the effect of all four proximate determinants of fertility. It is calculated by multiplying the four indices. Cm, Cc, Cfw, Ci are the indices of marriage, contraception, foetal wastage, and postpartum infecundability respectively. The indices can only take values between 0 and 1. When there is no fertility-inhibiting effect of a given intermediate fertility variable, the corresponding index equals 1, and if its fertility inhibition is complete, the index equals 0. These indices can be estimated from measures of the proximate variables and their estimates are shown below.

### Estimation of the index of marriage (cm)

The index of marriage measures the inhibiting effect of marriage on fertility in the population. It has to be noted that the higher the level of marriage in the population the lower the fertility inhibiting effect and the vice versa. The index equals one when all women are cohabitating, and zero when no women are in a union. The index of marriage is estimated using the formula;

3 $$ \mathrm{Cm}=\sum \mathrm{m}\ \left(\mathrm{a}\right)\ \mathrm{x}\ \mathrm{g}\ \left(\mathrm{a}\right)/\sum \mathrm{g}\ \left(\mathrm{a}\right) $$

Where

m (a) = age specific proportions currently married among females,

g (a) = age specific marital fertility rates.

m (a) is obtained by dividing the number of married women of a particular age group by the number of women in the same age group whereas; g (a) is calculated by dividing the births of a particular age group by the number of women in the same age group.

### Estimation of the index of contraception (cc)

The index of contraception in the model measures the inhibiting effect of contraception on fertility in the population. The higher the level of contraception in the population, the higher the inhibiting effect due to contraception and the lower the level of contraception the lower its inhibiting effect on fertility. The *Cc* equals one when no contraception is used, and zero when all fecund women use 100% effective contraception. The index of contraception is estimated using the formula

4 $$ {\displaystyle \begin{array}{l}\mathrm{Cc}=1\hbox{-} 1.08\ \mathrm{x}\ \mathrm{u}\ \mathrm{x}\ \mathrm{e}\\ {}\mathrm{e}=\sum \mathrm{e}\ \left(\mathrm{m}\right)\ \mathrm{x}\ \mathrm{u}\ \left(\mathrm{m}\right)/\mathrm{u}\end{array}} $$

Where;

u = Proportion using contraception among married women of reproductive age (15–49 years).

e = Average use effectiveness of contraception.

e (m) = method specific use effectiveness,

U (m) = proportion of women using specific method.

The coefficient 1.08 represents an adjustment for the fact that women do not use contraception if they know that they are sterile. The indices of use effectiveness proposed for particular contraceptives are; pill = 0.90, IUD = 0.95, sterilization = 1.00, injection = 0.99 and others = 0.70 [17]

### Estimation of the index of foetal wastage (Cfw)

The index of fetal wastage (Cfw) is defined as the ratio of the observed TFR to the estimated TFR with all fetal wastage which is (TFR + AFW), that is Cfw = TFR/TFR + AFW. Where AFW equals the average number of births averted per woman by the end of the reproductive years for all foetal wastage and which is estimated by

5 $$ \mathrm{AFW}=0.4\times \left(1+\mathrm{u}\right)\times \mathrm{TFW} $$

Where, TFW is the total foetal wastage rate and AFW is the average number of all foetal wastages per woman at the end of the reproductive years.

### Estimation of the index of postpartum infecundability

The index of postpartum infecundability measures the inhibiting effect of breastfeeding or abstinence on fertility in the population. The index of postpartum infecundability in the model is estimated using the effect of breastfeeding (lactation amenorrhea) or postpartum abstinence. The ratio of natural fertility in the presence and absence of postpartum infecundability therefore equals the ratio of the average birth interval without and with postpartum infecundability. If no breastfeeding and postpartum abstinence are practiced, the birth interval averages about 20 months, which is the sum of;

i). 1.5 months of minimum postpartum anovulation.
ii). 7.5 months of waiting time to conception.
iii). 2 months of time added by spontaneous intrauterine mortality.
iv). 9 months for a full term pregnancy.

Bongaarts Stated that, in the presence of breastfeeding and postpartum abstinence, the average birth interval equals approximately 18.5 months (7.5 + 2 + 9) plus the duration of postpartum infecundability [17]. The index of postpartum infecundability (Ci), therefore, is estimated as;

6 $$ \mathrm{Ci}=20/18.5+\mathrm{i} $$

Where

i = average duration of postpartum amenorrhea.

The index is calculated as the average birth interval in the absence of breastfeeding divided by the average length in the presence of breastfeeding. The index equals one in the absence of lactational amenorrhea or postpartum abstinence and declines in size as the period of postpartum infecundability rises.

## Results

### Estimated indices for the four major proximate determinants of fertility and their impacts

According to our analysis the value for Cm in 2011 was 0.622, where both delayed marriage and non-marriage inhibited fertility by 37.8%. In 2016 Cm was 0.656, in which case delayed marriage & non marriage inhibited fertility by 34.4%. The value for Cc in 2011 has been estimated to be 0.715, in which case contraceptive use has reduced fertility by 28.5%, while in 2016 the Cc was 0.693, where the use of contraceptives reduced fertility by 30.7% below its biological maximum of 15.3 births. The estimated values were presented in Table 1.

**Table 1:** Estimated indices for the four major proximate determinants of fertility, Ethiopia, EDHS 2011 and 2016

Estimated indices	2011	2016
TF	4.04	4.140
Cm	0.622	0.656
Cc	0.715	0.693
Ci	0.653	0.655
Cfw	0.908	0.908

Our analysis showed that the index for postpartum infecundity (Ci) in 2011 was 0.653, where lactation amenorrhea decreased fertility by 34.7%, and the index for Ci in 2016 was 0.693, where the effect of lactation amenorrhea reduced fertility by 34.5%. The indices for foetal wastages in both 2011and 2016 survey years were the same, i.e. 0.908. In both years foetal wastages inhibited 9.2% of the observed total births. The estimated values were presented in Table 1.

### Estimated indices for the four proximate determinants by selected background factors

The contribution of each of the four proximate determinants and their variability by these background variables in declining fertility were presented in Tables 2 and 3.

**Table 2:** Estimated Indices of the Proximate Determinants and Total Fertility Rates of the selected variables, Ethiopia, EDHS 2016

Selected variables	Indices of proximate determinants of fertility					
	Cm	Cc	Ci	Cfw	TF	TFR
Regions						
Tigray	0.648	0.639	0.590	0.888	15.3	3.319
Afar	0.752	0.881	0.660	0.946	15.3	6.329
Amhara	0.698	0.578	0.593	0.863	15.3	3.159
Oromia	0.708	0.711	0.612	0.921	15.3	4.341
Somali	0.760	0.984	0.746	0.962	15.3	8.211
Benishangul	0.748	0.714	0.570	0.966	15.3	4.499
SNNPR	0.687	0.598	0.588	0.908	15.3	3.356
Gambela	0.683	0.637	0.629	0.956	15.3	4.003
Harari	0.642	0.718	0.733	0.952	15.3	4.921
Addis Ababa	0.362	0.437	0.862	0.714	15.3	1.490
Dire Dawa	0.533	0.725	0.625	0.909	15.3	3.359
Wealth quintile						
Lowest	0.783	0.808	0.588	0.870	15.3	4.952
Second	0.760	0.694	0.581	0.914	15.3	4.285
Middle	0.713	0.633	0.583	0.899	15.3	3.619
Fourth	0.694	0.591	0.664	0.894	15.3	3.725
Highest	0.493	0.518	0.803	0.592	15.3	1.857
Education						
No education	0.311	0.694	0.580	0.709	15.3	1.358
Primary	0.608	0.590	0.637	0.766	15.3	2.678
Secondary	0.490	0.481	0.803	0.838	15.3	2.427
Higher	0.338	0.488	0.855	0.860	15.3	1.856
Residence						
Urban	0.428	0.496	0.826	0.575	15.3	1.543
Rural	0.640	0.668	0.592	0.659	15.3	2.552

**Table 3:** Estimated Indices of the Proximate Determinants and Total Fertility Rates of the selected variables, Ethiopia, EDHS 2011

Selected variables	Indices of proximate determinants of fertility					
	Cm	Cc	Ci	Cfw	TF	TFR
Regions						
Tigray	0.632	0.788	0.533	0.896	15.3	3.639
Afar	0.801	0.902	0.671	0.958	15.3	7.106
Amhara	0.688	0.655	0.542	0.888	15.3	3.318
Oromia	0.680	0.737	0.608	0.903	15.3	4.210
Somali	0.768	0.960	0.749	0.955	15.3	8.069
Benishangul	0.734	0.722	0.645	0.945	15.3	4.942
SNNPR(South Nations Nationalities and Peoples' Region)	0.574	0.738	0.581	0.908	15.3	3.419
Gambela	0.613	0.649	0.578	0.938	15.3	3.300
Harari	0.531	0.659	0.743	0.935	15.3	3.719
Addis Ababa	0.316	0.377	0.901	0.750	15.3	1.232
Dire Dawa	0.505	0.682	0.637	0.915	15.3	3.071
Wealth quintile						0.000
Lowest	0.756	0.865	0.548	0.869	15.3	4.765
Second	0.712	0.777	0.570	0.907	15.3	4.376
Middle	0.701	0.755	0.560	0.892	15.3	4.045
Fourth	0.682	0.679	0.645	0.881	15.3	4.026
Highest	0.512	0.481	0.697	0.628	15.3	1.649
Education						
No education	0.746	0.776	0.560	0.676	15.3	3.353
Primary	0.564	0.638	0.656	0.811	15.3	2.929
Secondary	0.371	0.428	0.897	0.838	15.3	1.826
Higher	0.409	0.351	0	0.838	15.3	0.00
Residence						
Urban	0.352	0.470	0.687	0.617	15.3	1.073
Rural	0.665	0.810	0.570	0.652	15.3	3.063

### Index of marriage (Cm)

In our study, in 2011 the highest Cm (0.801) was observed in Afar region whereas in 2016 its highest Cm (0.760) was shown in Somalia region. These showed that delayed marriage and non-marriage reduced fertility by 19.9% in 2011 in Afar and 24% in 2016 in Somalia regions. On the other hand, the lowest Cm (0.316 in 2011 and 0.362 in 2016) were demonstrated in Addis Ababa region, where delayed marriage and non-marriage reduced fertility by 68.4% in 2011 and 63.8% in 2016.

Regarding differential in residence, the Cm was highest among rural residents in both survey years where, Cm was 0.665 in 2011 and 0.640 in 2016. These showed that delayed marriage and non-marriage inhibited fertility by 33.5% in 2011 and 36% in 2016 among rural residents. Conversely, the Cm was lowest, 0.352 in 2011 and 0.428 in 2016, among urban residents. These showed that delayed marriage and non-marriage prevented fertility by 64.8% in 2011 and 57.2% in 2016 among urban women.

From the participants' education perspective, as per the study results, the Cm was highest, Cm = 0.746, among women with no formal education in 2011 and Cm = 0.608 among women who attended primary education in 2016. These indicated that delayed marriage and non-marriage inhibited fertility by 25.4% in 2011 and 39.2% in 2016 among women with no education and primary education, respectively. On the contrary, the Cm was lowest, 0.371, among women who attended secondary education in 2011 and 0.311 among women with no education in 2016. These showed that delayed marriage and non-marriage decreased fertility by 62.9% in 2011 among those who attended secondary education and 68.9% in 2016 among those who attended no formal education.

Differential of marriage indices with respect to differences in wealth quintiles of the women was analysed. Accordingly, the highest Cm, 0.756 in 2011 and 0.783 in 2016 was observed among participants in the lowest wealth quintile. These demonstrated that delayed marriage and non marriage prevented fertility by 24.4% in 2011 and 21.7% in 2016 among women with the lowest wealth quintile. The lowest Cm, on the other hand, 0.512 in 2011 and 0.493 in 2016 were realized amongst participants in the highest wealth quintile where delayed marriage and non-marriage averted fertility by 48.8% in 2011 and 50.7% in 2016.

### Index of contraceptives (Cc)

According to our analysis, in 2016, the Cc was highest, Cc **=** 0.984, in Somalia region and lowest, Cc = 0.437, in Addis Ababa region. These signify that contraceptive use inhibited only 1.6% of births in Somalia region whereas 56.3% in Addis Ababa region. The Cc was also the lowest, Cc = 0.377, in Addis Ababa in 2011 which shows that contraceptive use lowered fertility by 63.8%. Similarly, in 2011, the Cc was the lowest, Cc = 0.960, and contributed 4% in fertility reduction in Somalia region.

The Cc was analysed in relation to participants’ education level and variability has been observed. Accordingly, the lowest Cc, 0.351, was observed in 2011 among women having higher education and the lowest Cc, 0.481, was shown in 2016 among women with secondary education. These showed that the use of contraceptive lowered fertility by 64.9% in 2011 and 51.9% in 2016 among participants with higher and secondary education, respectively. On the other hand the highest Cc, 0.776 in 2011 and 0.649 in 2016, were observed among women with no formal education, which demonstrated that contraception decreased fertility by 22.4% in 2011 and 35.1% in 2016.

In our study, the Cc varied with respect to participants’ place of residence. Consequently, the highest Cc, 0.668 in 2011 and 0.810 in 2016 were observed among rural inhabitants whereas the lowest indices, 0.496 in 2011 and 0.470 in 2016 were demonstrated among their urban counterparts. These showed that contraceptive use in rural setting decreased fertility by 33.2% in 2011 and 19% in 2016 but it decreased fertility by 50.4% in 2011 and 53% in 2016 among the urban women.

In both surveys, the lowest Cc, 0.481 in 2011 and 0.518 in 2016, were observed among the participants in the highest wealth quintile. This showed that contraceptive use reduced fertility by 51.9% in 2011 and 48.2% in 2016. On the other hand, the highest Cc values, 0.865 in 2011 and 0.808 in 2016, were observed among participants in the lower wealth quintiles which indicated that contraceptive practices decreased fertility only by 13.5% in 2011 and 20% in 2016.

### Index of foetal wastage (Cfw)

The Cfw was calculated using the data from both surveys to detect any differences across the back ground variables. The Cfw was lowest, 0.750, in Addis Ababa and highest, 0.958, in Afar region in 2011. As a result, fertility declining effect of foetal wastage was 25% in Addis Ababa and 4.2% in Afar region.

The lowest Cfw, 0.628 in 2011 and 0.592 in 2016, were observed among participants in the highest wealth quintiles. Therefore, the effect of foetal wastage in reducing fertility was 37.2% in 2011 and 40.8% in 2016. Oppositely, the highest Cfw, 0.907 in 2011 and 0.914 in 2016 were demonstrated among participants in the second wealth quintile. These indicated that the effect of Cfw was 9.3% in 2011 and 8.6% in 2016 in reducing the total fertility.

The Cfw was lowest, 0.676 in 2011 and 0.709 in 2016, among women with no education, which showed that the effect of foetal wastage was 32.4% in 2011 and 29.1% in 2016 in fertility reduction. On the other hand, the highest Cfw values, 0.838 in 2011 and 0.860 in 2016 were demonstrated among participants having higher education. This showed that the fertility reducing effect of Cfw was 16.2% in 2011 and 14% in 2016 among women with tertiary education. The lowest values in Cfw, 0.617 in 2011 and 0.575 in 2016, were observed among urban residents, which indicated that the fertility declining effect of foetal wastage among urban women was 38.3% in 2011 and 42.5% in 2016. On the contrary, the highest Cfw, 0.652 in 2011 and 0.659 in 2016, were demonstrated among rural residents. These showed that the effect of foetal wastage in fertility reduction was 34.8% in 2011 and 34.1% in 2016 among rural residents.

### Index of postpartum infecundability (Ci)

In our analysis, the Ci varied across the participants’ background variables. Consequently, the highest indices, 0.901 in 2011 and 0.862 in 2016, of postpartum infecundability were observed in Addis Ababa region. This showed that the effect of Ci on total fertility decline was only 9.9% in 2011 and 13.8% in 2016 in the region. On the other hand, the lowest indices, 0.590 in 2011 and 0.590 in 2016 were observed in Tigray region which showed that the indices contributed 46.2% and 41% in fertility decline in 2011 and 2016 respectively, in the region.

Differences in Ci were also observed among the wealth quintiles where the lowest Ci values, 0.592 in 2011 and 0.581 in 2016, were observed among the lowest wealth quintiles. These demonstrated that the effect of Ci in fertility decline was 40.8% in 2011 and 41.9% in 2016 among the lowest wealth quintiles. The lowest indices, 0.560 in 2011 and 0.580 in 2016 were demonstrated among study participants with no formal education. These showed that the effect of Ci in declining fertility among participants with no formal education was 44% in 2011 and 42% in 2016. The highest Ci, 0.803, was observed among participants who attended tertiary education in 2011 whereas 0.897 among women who completed secondary education in 2016.

Lower indices of postpartum infecundability, 0.687 in 2011 and 0.570 in 2016, were shown among rural inhabitants. These represented that the effects of Ci in lowering fertility were 31.3% in 2011 and 43% in 2016 among rural women.

### Total fertility rate

Our analysis was inclusive of the calculation of the total fertility using revised Bongaarts model. As a result, the total fertility rate in 2016 was calculated to be 4.14 children per woman. The findings of our study also showed variability in TFR across the selected background variables. Accordingly, the highest TFR, 8.1 children per woman, and the lowest TFR, 1.5 children per woman, were observed in Somalia and Addis Ababa regions, respectively in 2016. In this year, the highest TFR of 4.9 children per woman was seen in lowest wealth quintile and the lowest TFR of 1.8 children per woman in the highest wealth quintile. In relation to the participants’ education, the highest TFR, 2.6 children per woman, and the lowest TFR, 1.8 children per woman, were observed among participants with primary education and higher education respectively in 2016. The TFR was 2.5 in rural and 1.5 in urban areas in this year.

### Projection of future fertility

The Bongaarts model of proximate determinants was used to project future fertility of the country. This was analysed by considering the expected contraceptive prevalence rate set by the Ethiopian government to attain a certain fertility level in 2020.

We supposed that TFR1 and TFR2 be the total fertility rates, in year 1 (the present year) and year 2 (year in the future), and the corresponding levels of contraceptive prevalence and use effectiveness be u1 and e1, and u2 and e 2, respectively. We calculated the level of total fertility for the changes of contraceptive uses from year 1 to year 2. However, we assumed that the other proximate determinants except for contraception remain constant, that is Cm1 = Cm2, Ci1 = Ci2 and Cfw1 = Cfw2. It is also assumed that contraceptive use-effectiveness remains constant.

Then TFR1/TFR2 = C1/C2 or

TFR2 = Cc2/Cc1 x TFR1 [19]

TFR2 =1–1.08xux e2/1–1.08x u1 x e1 x TFR1

In Ethiopia, contraceptive prevalence is 36% in 2016 and the government has planned to increase the contraceptive prevalence rate to 55% in 2020 [20].

The projected total fertility rate becomes

$$ {\displaystyle \begin{array}{l}\mathrm{TFR}2020=\frac{1-{1.08}^{\ast}\mathrm{U}{2020}^{\ast}\mathrm{E}2020}{1-{1.08}^{\ast}\mathrm{U}{2016}^{\ast}\mathrm{E}2016}\ \mathrm{X}\mathrm{TFR}2016\\ {}\mathrm{TFR}2020=\frac{1-1.08\mathrm{x}0.55\mathrm{x}0.933}{1-1.08\mathrm{x}0.36\mathrm{x}0.933}\mathrm{X}\ 4.6\end{array}} $$

Therefore, the projected TFR in 2020 will be 3.2 children per woman.

## Discussion

Our study assessed the proximate determinants of fertility and the role of the selected socio-economic variables in influencing the proximate determinants and fertility in Ethiopia. The revised Bongaarts model was used to determine the effects of proximate determinants on total fertility rate and its variability among the selected background variables. Generally, the results in this study showed that the impact of the four proximate determinants of fertility varies considerably among the selected background characteristics. In 2011, the Cm inhibited fertility by 37.8% whereas in 2016 it inhibited by 34.4%. Consequently, the Cm in 2011 contributed more for the reduction of fertility than Cm in 2016. This variation could be due to the fact that the proportion of married women was larger, 65% in 2016, when compared to 58% in 2011. This could be explained by the fact that the more women of child bearing age engage in marriage, the lower is its fertility inhibiting effect.

Both in 2011 and 2016, Cm played an important role in inhibiting fertility. This is in line with study done in Zambia, which showed marriage as the largest inhibitor on natural fertility [9]. The finding from Ghana also indicated a woman’s marriage status as important predictors of fertility outcomes [11]. Studies done in Sub-Saharan Africa identified non-marriage as attributable factor to decline fertility [12, 13]. Marriage was seen as the fertility inhibitor in Namibia and in Ethiopia [21, 22].The proportion of births inhibited due to the effect of contraception is higher in 2016 (30.7%) when compared to what was seen in 2011 (28.5%). This difference in the inhibition of fertility could be explained by the differences in the contraceptive prevalence rate. The contraceptive prevalence was higher (35%) in 2016 as compared to what was observed in 2011 (29%).e..

In current study, contraception practice played a role in influencing fertility. Study conducted in Bangladesh indicated that contraceptive practice played a key role in fertility change [8]. Contraceptive use also played an important role in explaining the fertility differentials in Philippines [10]. The finding from Ghana, Namibia and Ethiopia indicated that a woman’s contraceptive behavior as important predictors of fertility outcomes [11, 21, 22]. The contribution of contraception in inhibiting fertility in current study is higher than study done in Zambia, which showed that contraception use accounted for only 3% in inhibiting fertility [9]. The proportion of births prevented due to the effect of postpartum infecundability was slightly higher in 2011 (34.7%) as compared to what was in 2016 (34.5%). Postpartum infecundability is the second important proximate determinants of fertility both in 2011 and 2016. This is similar with the findings of Ghana, which indicated that postpartum infecundability was an important predictors of fertility outcomes [11]. Study done in Sub-Saharan Africa, identified post-partum amenorrhea being an important proximate determinant of fertility, which can be substantially extended by prolonged breastfeeding [13]. Study conducted in Namibia and Ethiopia identified postpartum infecundability as an important fertility inhibitor [21, 22].

In 2016 survey year the contraceptive use inhibited only 1.6% of births in Somalia region whereas 56.3% in Addis Ababa region. This huge difference observed between the two regions could be explained by variations in contraceptive prevalence rate between the regions, 55.9% in Addis Ababa and 1.5% in Somalia. Moreover, the variation in education among the study participants between the regions could have brought changes in awareness which in turn might have influenced the contraceptive use. Similarly, the highest and the lowest TFR were observed in Somalia and Addis Ababa region, respectively, in 2016. Study conducted in Ghana showed region of residence as predictor of fertility [11].

In 2016, the highest TFR was seen in lowest wealth quintile. Study done in Ghana and Philippines, showed wealth index as predictor of total fertility [10, 11].

In relation to the participants’ education, the highest TFR and the lowest TFR were observed among participants with primary education and higher education respectively in 2016. This is in line with study conducted in Ghana, which showed higher education levels are consistently associated with lower fertility rates [11]. Study done in Sub-Saharan Africa identified level of education as influencing factor of fertility [13]. In Ethiopia, main differences were observed across educational status [22].

The TFR was lower in urban areas than rural. This is in line with Ghanian study, in which urban residence is associated with lower fertility rates [11]. Study done in Sub-Saharan Africa identified place of residence as influencing factor of fertility [13]. In Ethiopia, main differences were observed across residential environment [22].

### Limitation

Reporting and recall bias particularly for age or other retrospective data relying in memory of the past event is the limitation of the study.

## Conclusion

Among the four proximate determinants of fertility, index of marriage was the most important inhibiting factor of fertility in 2011. On the other hand, postpartum infecundability was the first important fertility inhibitor in 2016. The contribution of the index of contraceptive in inhibiting fertility increased from 28.5% in 2011 to 30.7% in 2016. The index of foetal wastage contributed the least in both 2011 and 2016 survey years. The study also showed substantial differences in fertility inhibition by background factors in both 2011 and 2016.

The contribution of index of contraceptive in inhibiting fertility rate has not been as expected when compared to the contribution of other proximate determinants. The total fertility rate has shown variations across the regions. Therefore, the government and development partners need to design effective strategies and act in a concerted way to increase the prevalence of contraceptive use across the country, with particular emphasis to infrastructure limited regions.

The contribution of Ci in inhibiting fertility in 2016 was the highest. This calls for further strengthening of breast feeding as a good behavioural practice to promote natural family planning practices among breast feeding women..

## Abbreviations

AFW: All Fetal Wastage
Cc: Index of Contraceptive
Cfw: Index of Foetal Wastage
Ci: Index of Infecundability
Cm: Index of Marriage
EDHS: Ethiopian Demographic and Health Data
IUD: Intrauterine Device
TFR: Total Fertility Rate
TFW: Total Foetal Wastage
